# Genomic Characterization of the Barnacle *Balanus improvisus* Reveals Extreme Nucleotide Diversity in Coding Regions

**DOI:** 10.1007/s10126-021-10033-8

**Published:** 2021-05-01

**Authors:** Magnus Alm Rosenblad, Anna Abramova, Ulrika Lind, Páll Ólason, Stefania Giacomello, Björn Nystedt, Anders Blomberg

**Affiliations:** 1 Deparment of Chemistry and Molecular Biology, University of Gothenburg, Gothenburg , Sweden; 2 Department of Cell and Molecular Biology, National Bioinformatics Infrastructure Sweden, Science for Life Laboratory, Uppsala University, Husargatan 3, 752 37 Uppsala, Sweden; 3 Department of Biochemistry and Biophysics, National Bioinformatics Infrastructure Sweden, Science for Life Laboratory, Stockholm University, Box 1031, 17121 Solna, Sweden

**Keywords:** Genome, Barnacle, *Balanus improvisus*, Nucleotide diversity, Octopamine receptor

## Abstract

Barnacles are key marine crustaceans in several habitats, and they constitute a common practical problem by causing biofouling on man-made marine constructions and ships. Despite causing considerable ecological and economic impacts, there is a surprising void of basic genomic knowledge, and a barnacle reference genome is lacking. We here set out to characterize the genome of the bay barnacle *Balanus improvisus* (= *Amphibalanus improvisus*) based on short-read whole-genome sequencing and experimental genome size estimation. We show both experimentally (DNA staining and flow cytometry) and computationally (*k*-mer analysis) that *B. improvisus* has a haploid genome size of ~ 740 Mbp. A pilot genome assembly rendered a total assembly size of ~ 600 Mbp and was highly fragmented with an N50 of only 2.2 kbp. Further assembly-based and assembly-free analyses revealed that the very limited assembly contiguity is due to the *B. improvisus* genome having an extremely high nucleotide diversity (π) in coding regions (average π ≈ 5% and average π in fourfold degenerate sites ≈ 20%), and an overall high repeat content (at least 40%). We also report on high variation in the α-octopamine receptor OctA (average π = 3.6%), which might increase the risk that barnacle populations evolve resistance toward antifouling agents. The genomic features described here can help in planning for a future high-quality reference genome, which is urgently needed to properly explore and understand proteins of interest in barnacle biology and marine biotechnology and for developing better antifouling strategies.

**Supplementary Information:**

The online version contains supplementary material available at 10.1007/s10126-021-10033-8.

## Introduction 

Barnacles are sessile marine crustaceans encompassing around 1200 species that are usually gregarious and can be found at high densities in shallow and tidal waters around the globe (Newman and Abbott 1980). Barnacles play key roles in the marine ecosystem of intertidal rocky shores (Little et al. 2009). However, they can also establish populations in the deep-sea down to 2000 m (Araya and Newman 2018). Many barnacle species are epibionts on various marine animals including turtles and whales (Hayashi et al. 2013), where they are filter feeders and have a commensal relationship with their host. In contrast, the recently discovered parasitic barnacle *Anelasma* is found on the deep-sea lantern shark family Etmopteridae, where it embeds root-like structures into the flesh of the shark and parasitizes its host (Rees et al. 2014). Barnacles are suspension feeders that play critical roles in the regulation of primary production and the benthic-pelagic coupling of nutrients and organic matter, with zooplankton being an important food source for barnacles (Richoux et al. 2014). As a suspension feeder, they also impact on the water turbidity, which has many ecological implications. Further ecosystem services of barnacles include being prey for other organisms, e.g., carnivorous snails like *Nucella* see barnacle as an easy and less energetically demanding food (Dernbach and Freeman 2015).

Barnacles were in many respects the first model organism in evolutionary biology as reflected in Darwin’s work, with their specialized morphologies and reproductive systems making them ideal for testing theories on biological evolution (Hoeg and Moller 2006). However, barnacles are also of importance for diverse aspects of marine biotechnology. They are one of the main biofouling organisms on man-made underwater constructions, such as the piping of cooling units and ship hulls, causing considerable ecological and economic impacts. The global cost of marine biofouling is estimated to several billion US dollars annually (Schultz et al. 2011). Furthermore, barnacles produce one of the strongest underwater adhesives (Kamino 2006; Berglin et al. 2001) and are therefore of great interest in many applications.

The bay barnacle *Balanus improvisus* (= *Amphibalanus improvisus*) is a common barnacle species in temperate waters. Because of its extraordinary tolerance to low salinity (Sundell et al. 2019), it is used as a model organism to study osmoregulation in crustaceans and for studies of biofouling mechanisms in both marine and brackish conditions. This species is also well utilized as an experimental model since all-year-round culturing of *B. improvisus* has been developed, with the establishment of adult cultures on panels that provide a constant supply of barnacle larvae (Jonsson et al. 2018). Easy access to larvae makes it ideal as an experimental model for settling biology as well as antifouling research. *B. improvisus* is a truly cosmopolitan species and one of the most successful aquatic invaders worldwide. Genetic analysis showed that haplotypes were shared among populations on a global scale, indicating that long-distance dispersal through shipping has most likely played an important role in shaping its population genetic structure (Wrange et al. 2016).

Substantial amount of knowledge has been accumulated over the years regarding evolution, settlement, biotechnological aspects, and general biology of this species. However, there is an apparent lack of genomic data. While the Earth Biogenome project (www.earthbiogenome.org) aims for megabase contig N50 assemblies, there is currently only one draft genome with an N50 > 100 kbp published for barnacles, which is that of the acorn barnacle *Amphibalanus amphitrite* (Kim et al. 2019). Transcriptome sequencing provides datasets at a reasonable cost to be used in functional studies and for the identification of interesting genes (Lind et al. 2017; Lin et al. 2014). However, it is not suited to gain a complete view of the proteome since gene expression is dependent on life-stage, environmental condition, and tissue investigated. In addition, gene expression studies using transcriptome sequencing with short sequencing-reads on organisms that lack a reference genome is also unsatisfying in that it requires the generation of a de novo assembly. These short-read transcriptome assemblies are usually error prone, resulting in artificial chimeric sequences representing mixed isoforms and/or paralogous sequences, several partial transcript sequences for the same mRNA, as well as generally an overestimation of the total number of transcripts. Such errors might lead to erroneous interpretations of functional and evolutionary studies.

We here provide genome characteristics of *B. improvisus* in an attempt to establish a future high-quality reference genome. Besides setting the scene for future genome projects on *B. improvisus*, we report important insights into the extreme nucleotide diversity in the coding regions of this species, which will have consequences for applications in both marine biofouling and marine biotechnology.

## Materials and Methods

### Genomic DNA Preparation

High-quality genomic DNA was prepared from one adult individual of *B. improvisus* reared at the culturing facility at Tjärnö Marine Laboratory (University of Gothenburg, Strömstad, Sweden) that maintains cultures of the local population collected from outside the marine station (Jonsson et al. 2018). The DNA isolation procedure was performed as described earlier (Panova et al. 2016), except that sonication was used instead of homogenization with a pestle. Several DNA preparation kits were tried: the E.Z.N.A. Mollusc DNA Kit (Omega Bio-tek), the CTAB method (Winnepenninckx et al. 1993), the DNAzol kit (Life Technologies), and the E.Z.N.A. Blood DNA Mini Kit (Omega Bio-Tek, Norcross, GA), where the E.Z.N.A Blood DNA mini Kit produced the best results, and DNA prepared with this kit was used for sequencing. Adult *B. improvisus* individuals are rather small and have a maximum wet weight of about 12 mg, limiting the amount of total DNA per individual to ~ 10–15 µg.

### Sequencing and Assembly

The genomic DNA was used to construct two paired-end DNA libraries with average insert sizes of 150 and 245 bp, and these were sequenced by Illumina HiSeq20 00 (2 × 100 bp) to a total of 157 Gbp [sequencing was performed by the National sequencing facility SciLifeLab (Stockholm)]. The amount of DNA from one adult individual was unfortunately too low to produce additional long-insert sequencing libraries without PCR amplification, which is recommended for short-read assembly strategies. The reads were filtered and trimmed using the Fastx-toolkit v 0.0.13.2. Overlapping reads from the 150 bp library were joined using Flash (Magoc and Salzberg 2011), and reads were subsequently error-corrected with Quake v 0.3 (Kelley et al. 2010). De novo genome assembly was performed with CLC v 4.0.6 (CLCbio, Aarhus, Denmark), using *k*-mer = 27, a bubble size of 100 bp, and a cutoff = 200 bp for contig length. Different *k*-mer settings (27, 49 and 59) produced similar results.

The reads were mapped back to the assembly using BWAmem v 0.7.5a (Li and Durbin 2009), and the read coverage, GC content, and SNP frequency for each contig were calculated using qaCompute (Costea 2019), GATK v 2.4 (DePristo et al. 2011) and custom scripts. Inspection of the coverage vs GC plots indicated a subset of outlier contigs clearly representing bacterial genomes from *Flavobacteriales* (Bacteroidetes), e.g., top blastn hit was to *Lacinutrix* sp. 5H-3–7-4, a bacterium reported from subseafloor sediments, and *Sphingomonadales* (Alphaproteobacteria), which were removed from the assembly. Further contamination screening was performed using Deconseq v 0.4.3 with human, bacteria, archaea, and virus databases (Schmieder and Edwards 2011), as well as blastn to the vector sequences in UniVec (Kitts et al. 2011). Finally, all low-coverage contigs (< 10 ×) were excluded from the assembly. In total, 20 Mbp were removed. The unscaffolded assembly is accessible at the SciLifeLab repository (https://doi.org/10.17044/scilifelab.14339153).

To aid gene identification, the initial genome assembly was scaffolded, firstly by using the 245 bp paired-end genomic reads using BESST v 1.0.4.4 (Sahlin et al. 2014) and secondly by transcriptome scaffolding with the open reading frames extracted from the transcriptome assembly (see below) using ABySS (Simpson 2014). The alignment of transcript was done with BWA-mem (https://arxiv.org/abs/1303.3997), which handles heterozygosity well.

### Transcriptome Sequencing and Assembly

A cDNA-library was prepared from RNA extracted from the whole body of one single adult (another individual than used for DNA extraction), also taken from the culturing facility at Tjärnö Marine station (see above), using TruSeq mRNA sample kit (non-stranded). The cDNA library was sequenced by Illumina HiSeq2000 to get approximately 140 million paired-end reads (2 × 100 bp). After trimming using cutadapt v 1.2.1 (Martin 2011) and filtering using the Fastx-toolkit v 0.0.13.2, 109 million read-pairs and 186 million singlets remained. These were assembled by Trinity (r2013_08_14) (Haas et al. 2013) by first applying digital normalization using default parameters (K25, C50, pctSD100) producing 77,585 “genes” with a total of 158,153 isoforms (“transcripts”). A total of 92,295 open reading frames were predicted from this transcriptome assembly by TransDecoder v 2.0 . By homology searches (blastp, e-value < 1E − 10) of the *Drosophila melanogaster* proteome (FlyBase annotation release 5.55), we identified a non-redundant benchmark set of 4705 putatively full-length *B. improvisus* conserved proteins denoted as “complete” by Transdecoder.

### Nucleotide Diversity of Coding Genes

To investigate the general level of nucleotide diversity in coding regions, we searched the genome assembly for conserved putative single-copy genes where the two alleles are found on separate contigs in the assembly process. First, we used CEGMA 2.5 to align a set of 458 conserved single-copy protein-coding genes to the assembly, resulting in the identification of 336 predicted partial or complete proteins. Short proteins (< 300 aa) were removed, resulting in a set of 137 *B. improvisus* proteins with good hits to CEGMA proteins. Each protein was aligned back to the assembly using Scipio v 1.4.1 (Keller et al. 2008), with a relatively stringent Scipio score cutoff (*–min_score 0.50*) and with hits restricted to single contigs (*–single_target_hits*). The 70 alignments that displayed exactly two high-scoring alignments (i.e., putative alleles) were manually inspected to rule out alignment artifacts, leading to the exclusion of four genes from the analyses of nucleotide diversity due to potential frameshift errors, i.e., the nucleotide diversity was calculated based on 66 alignments/genes. The violin plot for the distribution of the diversity was made using ggplot2 v 3.3.3 in R with the functions geom_violin and geom_jitter with the following settings, width = 5 and height = 7.

### Nucleotide Diversity of the α-Octopamine Receptor OctA

Adults and cyprids from a *B. improvisus* population cultured at the Tjärnö marine station (see above) were analyzed for the nucleotide diversity of the octopamine receptor OctA. In short, rapid amplification of cDNA ends (RACE) was used to get the sequences up-stream and down-stream of the OctA CDS. From the RACE-product sequences, the up-stream/forward (fw) and down-stream/reverse (rev) PCR primers were constructed: R0-fl_fw GCTGTGTAGAGCTGTGACTGAC (≈ 150 nt upstream start-ATG) and R0-fl_rev GCCGGACTGGACTCCTGCTC (107 nt down-stream of stop-TGA). For more details on the PCR amplification procedure, see Lind et al. 2010 (Lind et al. 2010). PCR amplicons were sequenced by Sanger sequencing (MWG Biotech). For four adult individuals, we cloned the OctA receptor and sequenced a number of clones to identify both alleles (adult 1: 6 clones; adult 2: 25 clones; adult 3: 7 clones and adult 4: 7 clones). The sequences for the clones were aligned individual-wise, and the sequences were manually curated to select one sequence each for the two alleles in each individual. For the population-based data, the sequences originate from a mix of individuals (either adult or cyprid individuals) or from cyprid batches where each batch contains several hundreds of cyprids. For these populations, a lower number of clones were generated and sequenced per sample, with an average of 1.5 clones per individual/batch (excluding the adults for which we had a large number of clones). The cDNA sequence population data is obtained from 17 adults (22 sequences) and from a batch of cyprids (2 sequences), whereas the genomic sequence population data is obtained from 4 adults (8 sequences, same adults as mentioned above), 3 single cyprids (3 sequences), and from a batch of cyprids (4 sequences). DNA sequences of the CDS (from start to stop) were aligned using ClustalW, and the average pairwise nucleotide diversity per site or per fourfold degenerate site for any two sequences (π and π4D, respectively) for the coding region was calculated. Substitution rates were estimated from pairwise codon alignments (Nei and Gojobori 1986) as implemented in PAML v 4.9e. Tajima’s D for the coding regions of both alleles of adult 1–4 was calculated using the R package PopGenome (v 2.7.5). Loading the alignment into GENOME.class was followed by neutrality.stats and get.neutrality. The alignment of 1473 positions contained 116 segregating sites of 1463 valid sites.

### Repeat Content

The repeat content of the genome assembly was estimated based on homology search to known Arthropoda repeats as implemented in RepeatMasker v 4.0.1 . To increase sensitivity, we also constructed a species-specific *B. improvisus* repeat library, using RepeatExplorer (Novak et al. 2010, 2020) with a small subset of 200,000 randomly selected sequencing reads as input, corresponding to about 0.03 × coverage of the *B. improvisus* haploid genome size. The RepeatExplorer pipeline is based on the logic that similarity-hits in the read-read comparison of a shallow-coverage subset of reads occur almost exclusively between reads from repetitive sequences.

As an additional independent analysis of the genomic repeat content, we also interrogated the *k*-mer frequency spectra of the raw sequencing reads.

### Genome Size Calculation Using *k*-mer Histograms.

Jellyfish (v 1.1.11 and 2.1.4) was used to create *k*-mer histograms, and the genome size was calculated using the following formula: Genome size = *n* * (L-k + 1)/C, where *n* = total number of reads, *L* = average read length, *k* = *k*-mer size, and *C* = coverage in homozygous *k*-mer peak.

### Genome Size Experimental Determination.

For flow cytometric measurements of nuclear DNA content, dissected adult barnacle cirri and whole cyprid larvae were frozen at − 80 °C. Nuclei preparation and DNA staining were performed following guidelines earlier outlined (Hare and Johnston 2011). In short, a pool of frozen cirri from 30 adults and a pool of 30 larvae were thawed on ice and immediately processed. Samples were gently homogenized in 1 ml of Galbraith buffer (Hare and Johnston 2011) with a pestle (15 strokes). Each nuclei suspension was prepared by filtering through a 20-μm nylon net filter (Millipore) to remove cellular debris. Chicken erythrocytes were used as a genome size standard (C-value corresponding to 1.14 Gbp) as earlier described (Dhillon et al. 1977). Fresh chicken blood was mixed with blood anticoagulant citrate dextrose (ACD) solution and stored at − 20 °C. Chicken blood (15 μl) was added to 1 ml of Galbraith buffer, followed by homogenization and filtering procedures as described above for the barnacle samples. Aliquots of chicken nuclei were added to freshly prepared nuclei suspensions from barnacle cirri and larvae. Nuclei were stained by adding propidium iodide (50 μg/ml) together with RNase (20 μg/ml) (to prevent staining of double-stranded RNA) followed by incubation in the dark at 4 °C for roughly 22 h. The mean fluorescence of nuclei in each sample was quantified in at least three replicates using a BD FACSAria II flow cytometer (BD Biosciences). The cytometer was activated for the red fluorescence detection (at 615 nm). At least 10,000 nuclei were measured per sample. Data were analyzed with BD FACSDiva software, and DNA values were calculated by comparison to the chicken genome.

## Results 

### Genome sequencing and assembly

De novo genome assembly based on 157 Gbp short-read Illumina sequences from one adult individual of *B. improvisus*, which corresponds to > 200 × coverage (see genome size estimate below), resulted in 587,357 contigs > 200 bp in size (Table 1). The assembly was remarkably fragmented, with an N50 of 2.2 kbp and a total assembly size of ~ 600 Mbp. Contigs larger than 1 kbp summed up to a total of 449 Mbp, while only 22 Mbp (4%) of the assembly were represented in contigs longer than 10 kbp. Unfortunately, the DNA content from one adult individual did not suffice to also produce a long-fragment scaffolding library. Scaffolding, either with the longest fragment paired-end library or with transcript (cDNA) data, did only marginally improve the assembly (Table 1). The poor contiguity in the pilot assembly suggested that a future reference genome assembly effort will pose a considerable challenge. To create a good knowledge base for a future reference genome project, we set out to characterize the genome architecture in *B. improvisus* regarding genome size, repeat content, gene structure, and nucleotide diversity.

**Table 1:** Genome assembly statistics for *B. improvisus* The scaffolding was done first using the longer paired-end data and secondly using transcriptome sequences

	Unscaffolded (N50 = 2.2 kbp)	Paired-end scaffolding	Transcriptome scaffolding
Number of contigs/scaffolds^a^	587,357	545,974	530,331
Total size^a^	612 Mbp	612 Mbp	612 Mbp
Size in contigs/scaffolds^b^			
> 1 kbp	449 Mbp	465 Mbp	468 Mbp
> 5 kbp	119 Mbp	166 Mbp	192 Mbp
> 10 kbp	22 Mbp	42 Mbp	74 Mbp
Max contig/scaffold size^b^	39 kbp	40 kbp	82 kbp
Fraction of CEGMA core genes			
Complete (> 70% aligned)	48%	52%	61%
Partial (> 30% aligned)	84%	85%	92%

^a^ > 200 bp

^b^Excluding gaps

### Genome Size Estimation

No estimate has been published for the haploid genome size of *B. improvisus*. Highly variable genome sizes have been reported for other species of barnacles. Conflicting experimental estimates of 0.7 Gbp or 1.4 Gbp were presented for the haploid genome size of the closely related barnacle *Balanus amphitrite* (= *Amphibalanus amphitrite*) (Bachmann and Rheinsmith 1973; Rheinsmith et al. 1974). Two other acorn barnacles of the family Balanidae have larger haploid genome sizes of 1.4 Gbp (*Semibalanus cariosus*) and 1.26 Gbp (*Amphibalanus eburneus*) while the haploid genome sizes of the more distantly related species *Tetraclita rubescence* and *Pollicipes polymerus* are 2.6 Gbp and 0.9 Gbp, respectively (Gregory 2020; Bachmann and Rheinsmith 1973; Rheinsmith et al. 1974). Thus, genome size does not clearly follow phylogenetic classification. Given the fragmented assembly for *B. improvisus*, the total assembly size of ~ 600 Mbp might not reliably predict the genome size. We therefore experimentally determined the haploid genome size of *B. improvisus* by performing flow cytometry (FACS) using DNA-stained nuclei.

**Fig. 1:**
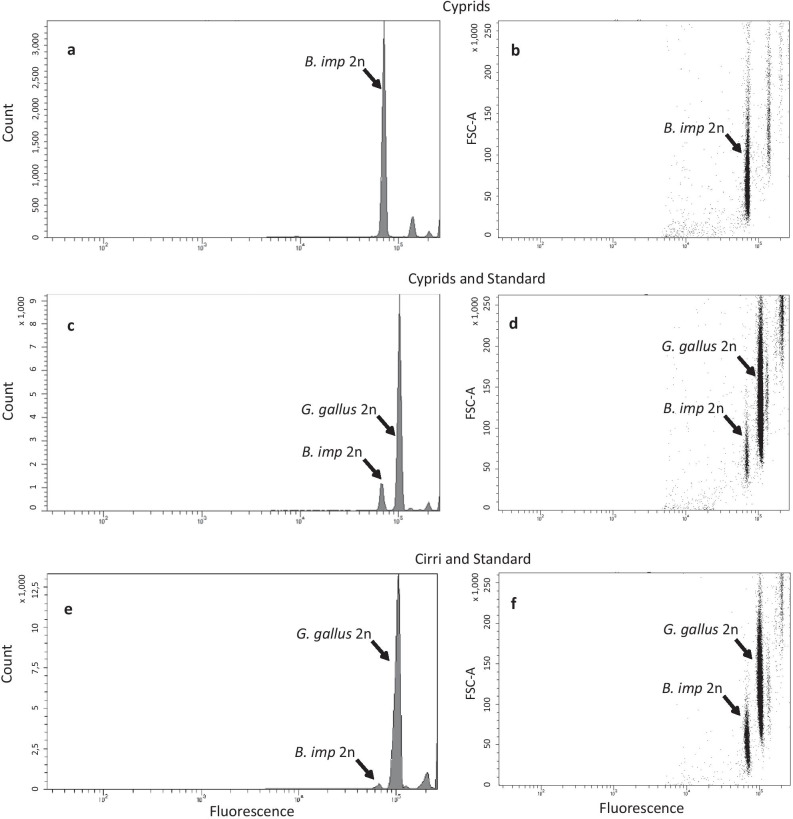
Experimental estimate of *B. improvisus* genome size. Flow cytometry spectra of propidium iodide-stained nuclei from cyprids and cirri of *B. improvisus* and chicken *G. gallus* (chicken used as a reference, C-value of 1.14 Gbp) are shown. (**a)** Histograms showing fluorescence intensity peaks of *B. improvisus* 2n nuclei extracted from cyprid larvae and (**b)** showing corresponding forward scatter light (FSC-A; reflecting size of nuclei/particles), in both cases in relation to fluorescence. (**c)** Fluorescence peaks of cyprid 2n nuclei and reference *G. gallus* 2n nuclei and (**d)** corresponding to FSC-A spectrum, indicating distinct barnacle and chicken nuclei populations. (**e)** Histograms showing fluorescence peaks of 2n nuclei extracted from adult barnacle cirri and chicken 2n nuclei, and **f** corresponding to FSC-A spectrum. For actual estimates of haploid genome size for each sample, see Table 2

We investigated both whole cyprids as well as cirri from adults to get estimates on DNA content from different life-stages (Fig. 1; Table 2). Isolated cirri were used from adults to minimize the risk of contamination by haploid germ-cells. Graphs representing fluorescence against scattered light [forward scatter (FSC); the larger the particle the higher the scatter) (Fig. 1b, d, and f)] indicated that the main fluorescence peak of *B. improvisus*, denoted with 2n in Fig. 1a, showed mainly a low-scatter pattern whereas the material of the other peaks scattered light to a greater extent. These other peaks were probably caused by larger particles, containing for example cell debris, multiple cells, or nuclei with adherent fluorescence (Hare and Johnston 2011), and were accordingly disregarded for the analysis of the genome size. Nuclei from chicken erythrocytes were used as a genome size standard (haploid genome size 1.14 Gbp), resulting in an estimated haploid genome size of *B. improvisus* of 738 Mbp ± 9 Mbp (SD) with insignificant differences in size estimates between cyprids or adult cirri (Fig. 1c and e; Table 2).

**Table 2:** Nuclear haploid DNA content of *B. improvisus*. The nuclear DNA content was obtained by propidium iodide staining and flow cytometry analysis. Chicken red blood cells (C-value 1.16 pg, corresponding to 1.14 Gbp) were used as a reference to calculate the *B. improvisus* haploid C-value, and the corresponding Mbp values according to the factor 1 pg = 978 Mbp (Gregory 2020)

Sample	Tissue	C-value*	Average C-value
Cyprid larvae	30 individuals	747.0 Mbp	738 ± 9.2 Mbp
Adult	Cirri	730.5 Mbp	

*Means for each tissue type generated from three technical replicates for each nuclei preparation (coefficient of variation for technical replicates ≈ 2%)

**Fig. 2:**
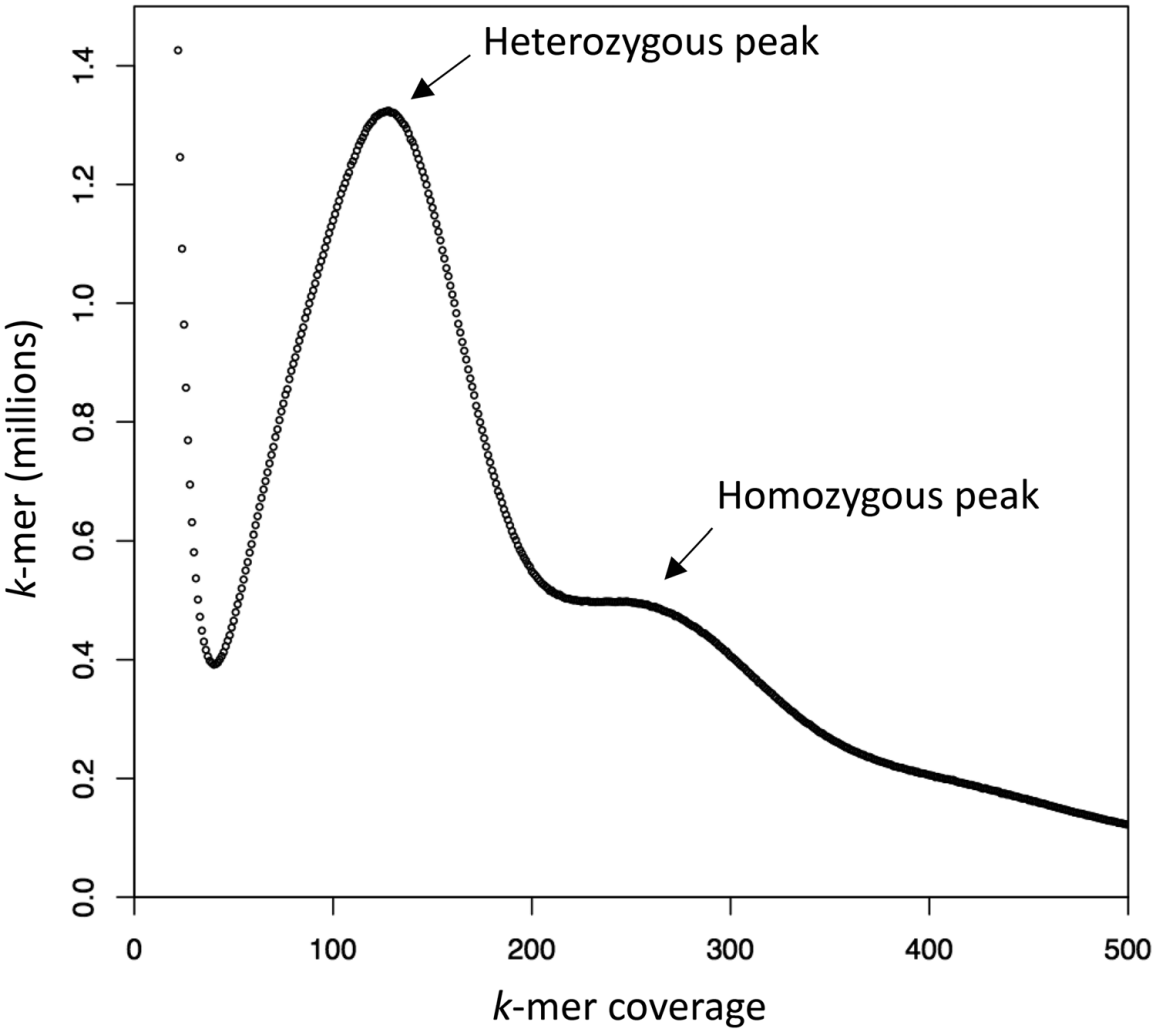
Computational estimate of *B. improvisus* genome size. The distribution of *k*-mer counts vs *k*-mer coverage of the *B. improvisus* pilot genome assembly, showing a pronounced heterozygous peak (≈125 k-mer coverage) and a much smaller homozygous peak at double *k*-mer coverage (≈250). *k*-mer = 15

We also investigated the *k*-mer frequency spectra of the unassembled genomic reads, using the total amount of *k*-mers and the average expected *k*-mer coverage to estimate the genome size. This resulted in a genome size estimate in the range 1.37–1.46 Gbp, which is deemed to reflect the diploid (2n) size since we could identify a small homozygous peak at twice the coverage (≈250) in comparison to the heterozygous peak (≈ 125) (Fig. 2). We conclude that the *B. improvisus* haploid genome is ~ 740 Mbp, and that our diploid assembly size based on short reads (~ 600 Mbp) therefore only represents 43% of the diploid genome.

### Repeat Content

Since longer repeats are expected to be collapsed in genome assemblies based on short reads and small insert sizes, it is nontrivial to estimate the true repeat content of the genome of *B. improvisus*, e.g., by using de novo repeat identifiers like RepeatModeler. We started by using RepeatMasker to align repeat libraries from other species to the genome assembly, resulting in only 1% of the assembly being marked as repeats. In line with this result, the corresponding RepeatMasker-analysis on the draft genome *A. amphitrite* yielded roughly 5% repeats (Kim et al. 2019). We then constructed a species-specific repeat library for *B. improvisus* using the computational pipeline RepeatExplorer that is tailored for the identification of high-copy repeats from short sequence reads (Novak et al. 2020). This shallow-coverage assembly resulted in 1700 repetitive sequences with a total size of 0.6 Mbp, representing complete or partial representations of high-copy repeat sequences. The size distribution of these repeats (median length 303 bp; min = 90 bp, max = 2361 bp; supplementary Fig. S1) clearly outline the challenge of properly assembling these repeats based on our short-read sequences (100 nt). Running RepeatMasker with the species-specific repeat library resulted in a much larger proportion (17%) of the genome assembly being indicated as repeats. The large difference in the estimated repeat content between the two methods indicated that a majority of the *B. improvisus* repeats are novel or very diverged compared to known repeats in other organisms. Since many repeats can be expected to be heavily underrepresented in our draft assembly, we also used the species-specific repeat library to analyze the raw genome sequencing reads. This analysis resulted in an estimated 40% repeat content. We also observed that 75% of the *k*-mers (*k* = 15) appeared at a coverage above 400 × , thus indicating that a large fraction of the genome contains repetitive sequences with high sequence similarity. Since the detection methods can be expected to be underestimating rather than overestimating the repeat content, we conclude that at least 40% of the *B. improvisus* genome is represented by high-copy repeat families of which most are novel.

### Gene Structure and Completeness

**Fig. 3:**
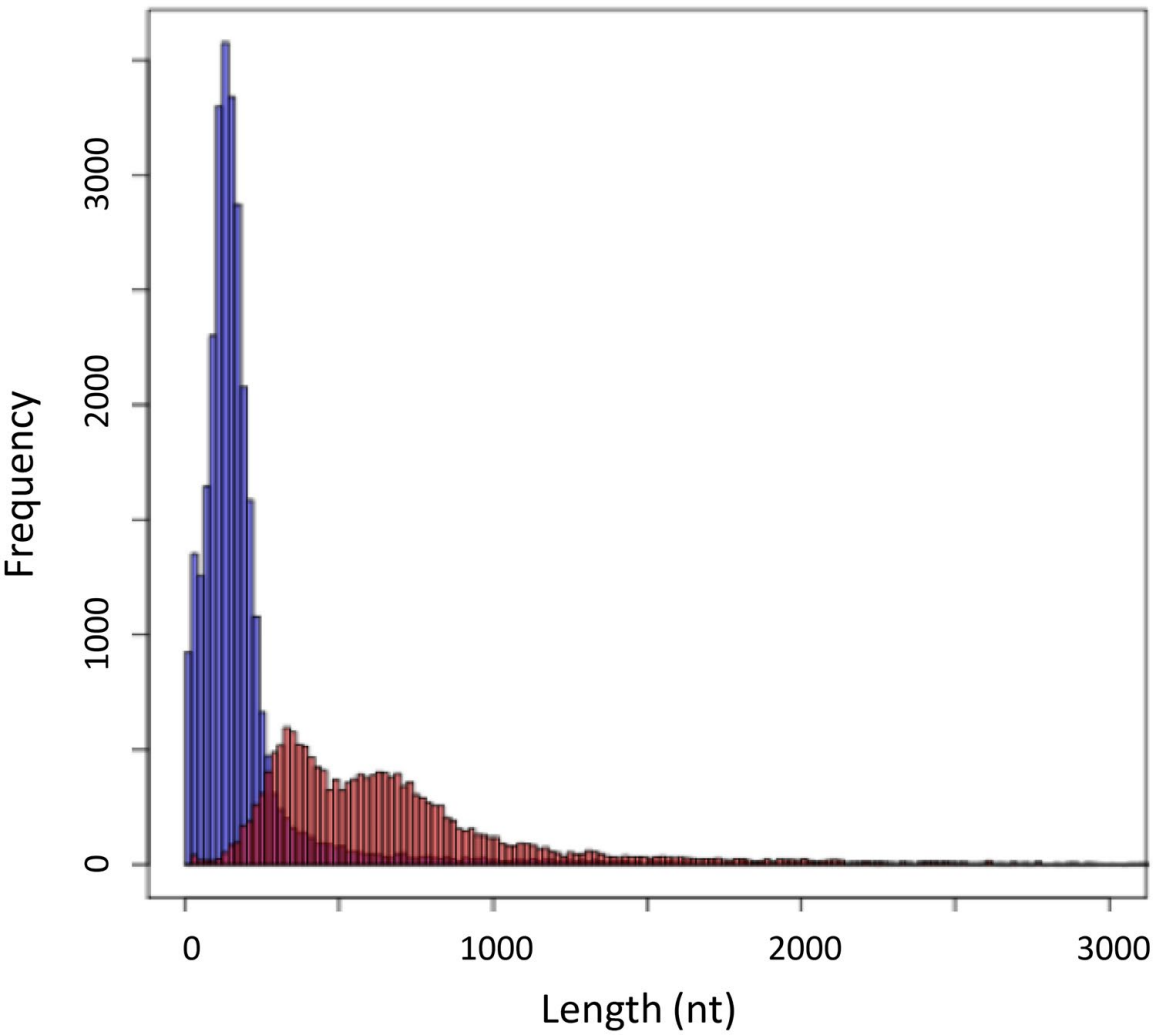
Size of exons and introns. Histogram of intron (red) and exon (blue) length based on gene structure predictions is shown from *B. improvisus* transcripts matching conserved genes from Drosophila and the assembly contigs. No minimum length cutoff was used. Bin-size 20 bp

To investigate the overall gene structure of *B. improvisus*, we constructed a non-redundant reference set of 4705 putative full-length transcripts from the *B. improvisus* transcriptome assembly, with clear protein homology to the *D. melanogaster* proteome. The reference transcripts were aligned to the genome assembly using Scipio, allowing transcripts to span across DNA contigs. Despite the very fragmented genome assembly, the coding gene content appeared to be reasonably well represented based on CEGMA core gene analyses (Parra et al. 2009); 61% of the genes were predicted to be completely recovered (> 70% aligned) within single scaffolds, and 92% were partially recovered (> 30% aligned) (Table 1). This enabled a first (although non-exhaustive) characterization of the *B. improvisus* gene structures. The *B. improvisus* genes typically displayed short exons (average 202 bp) (Fig. 3), which is in line with the average exon lengths reported for *A. amphitrite* of 280 bp (Kim et al. 2019). We estimated the intron size in *B. improvisus* to on average 706 bp, a number that is likely heavily underestimated since many genes contain intronic assembly gaps of unknown lengths, which cannot be properly accounted for in the current fragmented genome assembly. Based on some of the larger contigs, however, we concluded that introns of several thousand base-pairs were not uncommon.

### High Nucleotide Diversity in Coding Regions

**Fig. 4:**
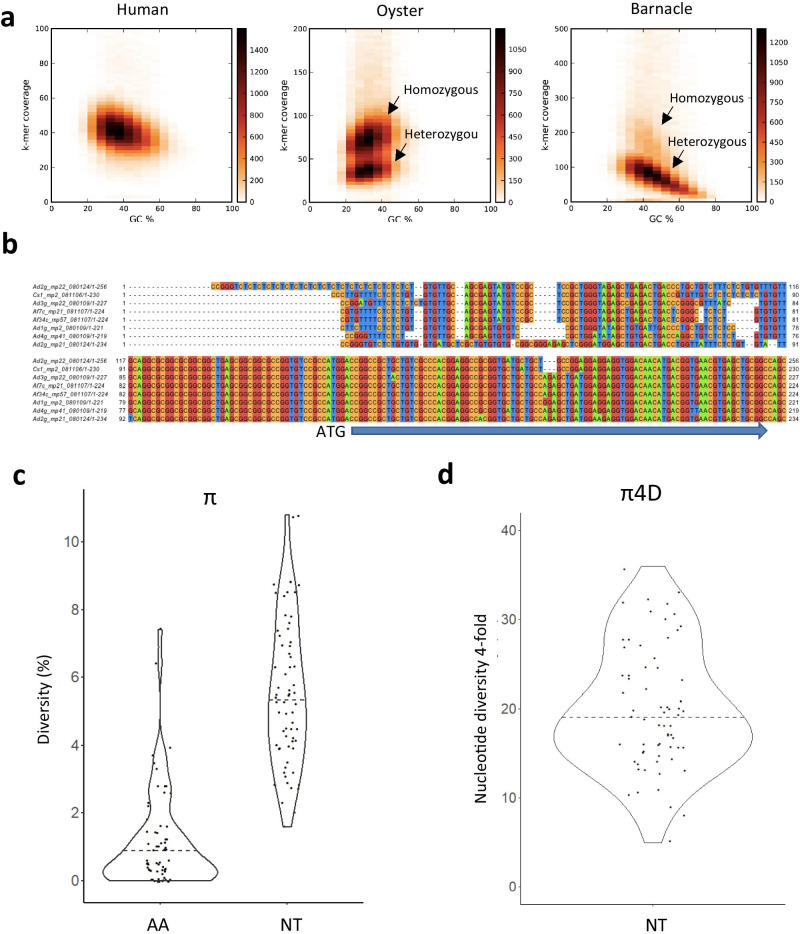
Genetic diversity in *B. improvisus*. (**a)** *K*-mer analysis and comparison of heterozygosity with other species. To compare the heterozygosity estimate from the *k*-mer analysis of *B. improvisus* genome to non-barnacle species, the preqc program in the sga assembly package (Simpson 2014) was used adding data for human and oyster (Zhang et al. 2012). The preqc plots the GC content against *k*-mer coverage (shown by color). Homozygous and heterozygous parts are indicated (in the human plot the heterozygous part is barely visible). (**b)** Example of 5′ variation in the alpha-octopamine receptor (OctA). Sanger-sequenced clones showing alleles from 7 individuals for the region up- and downstream of the start ATG. All sequences are consensus of at least two independent clones. The top and the bottom sequences are two alleles from the same adult (Ad2). Ad adult from laboratory culture, Af adult from field experiment, C cyprid sequence taken from a population. Numbers refer to the separate individuals. g = genomic, c = cDNA, mpX_xxxxxx = the clone id. The blue bar at the bottom indicates the coding sequence. (**c)** Sequence diversity from comparing either amino acid or nucleotide sequences in coding regions. Diversity was determined, from pairwise alignments of alleles in the *B. improvisus* genome assembly, using the 66 conserved core proteins from the CEGMA analysis. AA amino acids, NT nucleotides. Violin plots made in R, with dots representing the individual diversity values. (**d)** π4D indicate the pairwise genetic diversity for fourfold degenerative sites, based on the same set of 66 predicted core conserved proteins from the CEGMA analysis. Violin plot as in **c**

The *k*-mer frequency spectra from the raw sequence reads showed a pronounced bimodality at relatively short *k*-mers (*k* = 15: Fig. 2), a pattern that is typical for species with high heterozygosity (Messmer et al. 2018; Roach et al. 2018; Kim et al. 2019). In fact, the *k*-mer frequency spectra indicated that very few regions in *B. improvisus* are homozygous, since the homozygous peak in the frequency spectra was rather insignificant. The heterozygosity of *B. improvisus* was also compared by *k*-mer analysis to human and oyster and found to be much more extreme; for human, the heterozygous part is barely visible (Fig. 4a).

To further interrogate the tentatively very high nucleotide diversity, we first performed PCR cloning and Sanger sequencing of both alleles from an earlier reported single-copy gene without introns, the α-octopamine receptor OctA (Lind et al. 2010), in four *B. improvisus* adult individuals (Table 3). We found that the pairwise nucleotide diversity (π) in the coding region between alleles from a single individual in this 1473-bp single-exon CDS was in the range 2.6–3.9%. Analyzing genomic and cDNA clones of the OctA alleles from several adults and cyprid larvae from one single population outside the marine station at Tjärnö (Sweden), we estimated the average π in *B. improvisus* for the OctA CDS to be 3.6% (Table 3). Furthermore, by extending the sequence analysis to the 5′ UTR region of OctA, we identified an even higher sequence variation with multiple SNPs and several insertions and deletions (Fig. 4b).

**Table 3:** The nucleotide diversity in the α-octopamine receptor is shown. The pairwise nucleotide diversity between alleles in the 1473 bp single-exon CDS (coding sequence) of the α-octopamine receptor (OctA) within four single adults (genomic DNA), as well as between a mixture of alleles originating from genomic DNA or cDNA coming from both adults and cyprids. *π* nucleotide diversity for all nucleotides, *π4D* nucleotide diversity for fourfold degenerate sites, *dN* nucleotide diversity for non-synonymous sites, *dS* nucleotide diversity for synonymous sites

Sample individual/populations	Nucleotide divergence				
	π (%)	π4D^a^ (%)	dN^b^	dS^b^	dN/dS^b^
Adult 1	3.5	7	0.0057	0.069	0.083
Adult 2	3.9	12	0.0048	0.11	0.042
Adult 3	3.7	11	0.0069	0.12	0.057
Adult 4	2.6	6	0.0067	0.061	0.11
Population adult/cyprid genomic DNA (*n* = 15)	3.7^c^	ND	ND	ND	ND
Population adult/cyprid cDNA (*n* = 24)	3.6	ND	ND	ND	ND

*ND* not determined

^a^Fourfold degenerate sites. There are roughly 278 ± 1 (SD) fourfold degenerate sites in OctA, and these are used to calculate the neutral nucleotide variation

^b^dN (non-synonymous), dS (synonymous), and dN/dS are calculated according to Nei and Gojobori (1986). There are 1245 ± 14 non-synonymous sites and 186 ± 14 synonymous sites in the OctA sequences

^c^Note that this value is calculated also including the two alleles each of adult 1–4

To give an estimate of the general level of π in coding regions, we identified in the pilot genome assembly conserved putative single-copy genes (CEGMA genes) where the two alleles had been split up in separate contigs in the assembly process. Despite being presumably single-copy, most CEGMA genes aligned at two different assembly contigs (average 2.05 alignments per CEGMA gene), emphasizing the diploid nature of the assembly. In total, 66 proteins displayed exactly two high-scoring alignments in the assembly, putatively representing the two alleles. These 66 CEGMA genes displayed a median pairwise nucleotide diversity of 5.3% (range 2–11%) (Fig. 4c), which corresponds to an average pair-wise nucleotide diversity of 5.5% ± 2.1% (SD) (see supplementary Table S1 for the individual π values for the 66 CEGMA genes). Strikingly, even for the 16 proteins in this set with no amino acid mismatch between the alleles, the median nucleotide diversity was still 4.0% (π = 4.2% ± 1.6% (SD)).

The nucleotide diversity is often indicated for fourfold degenerate (π4D) sites, since changes in these will not involve any changes in amino acids and thus not be under selection at the protein level. The 66 CEGMA genes analyzed contained 13,135 fourfold degenerate sites of which 2688 were heterozygous, leading to an average π4D diversity of 20.1% ± 6.9% (SD) (median 19.0%: Fig. 4d; Table S1). The CEGMA genes had an average dN/dS value of 0.029 ± 0.035 (SD). The fact that dN/dS << 1 reflects the overall strong purifying selection on these highly conserved and mostly house-keeping core proteins (Parra et al. 2007). We also estimated the Tajima’s D statistics for the eight OctA alleles from the four adults to D =  − 0.80. We found the average π4D diversity for the α-octopamine receptor OctA of the four adult individuals to be 9.0% and the proportion of changes in synonymous sites (dS) to be in the same range (Table 3). Taken together, our data suggests an extraordinarily high heterozygosity in coding regions as a common feature in *B. improvisus*.

## Discussion

No barnacle high-quality reference genome is available despite the relatively small barnacle genome sizes of 655–2540 Mbp (Gregory 2020). We here provide an initial genome characterization of the biotechnologically important bay barnacle *B. improvisus*, regarding genome size, repeat content, nucleotide diversity, and gene structure. Despite the fact that our pilot genome assembly is highly fragmented, such an assembly can be very useful in order to investigate specific genes and gene families (Lind et al. 2010, 2013, 2017; Abramova et al. 2019). It is also an important resource in designing future genome projects aiming for a high-quality reference genome.

There are currently draft genomes published for two species of barnacles. For the acorn barnacle *Semibalanus balanoides*, short-read (Illumina) sequencing of genomic DNA from a pool of 60 individuals resulted in a highly fragmented assembly of (N50 = 250 bp) (Flight and Rand 2012). The contigs of this *S. balanoides* draft genome summed up to 31 Mbp, which is only about 2% of the expected haploid genome size clearly indicating the challenge with assembling this genome. An improved version of the *S. balanoides* genome was recently released (Nunez et al. 2020b). This updated draft genome is based on a combination of short-read (Illumina) and long-read (PacBio) technologies (one individual sequenced with each technology) with all reads combined in the final assembly (N50 = 56 kbp). Two individuals were used because too little DNA could be extracted from a single individual, in line with our experience with *B. improvisus* (Panova et al. 2016). This updated *S. balanoides* assembly totaled to 486 Mbp, with the predicted haploid genome size being 1.05–1.44 Gbp, indicating missing parts. The lack of genome material in the final assembly is also suggested by the fact that ≈ 30% of BUSCO genes are missing.

For the barnacle * A. amphitrite*, a draft genome based on long-read (PacBio) sequences was recently published with N50 = 240 kbp (Kim et al. 2019). The *A. amphitrite* genome was estimated to 481 Mbp based on *k*-mer analysis of sequence reads, while the total genome assembly summed to 609 Mbp. Both these numbers are below the experimental size estimate of the haploid genome of *A. amphitrite* that is in the range 0.7–1.4 Gbp (Gregory 2020; Bachmann and Rheinsmith 1973; Rheinsmith et al. 1974). This *A. amphitrite* draft genome was made from DNA of pooled tissues from two individuals, once again because of the challenge to isolate enough DNA from one single individual (Schultzhaus et al. 2019). With the reported high heterozygosity also in this species (Kim et al. 2019), the pooling of two individuals should result in four haplotypes providing a challenge for the assembly process. A possibly non-negligible amount of allelic contigs in the assembly is indicated by the rather high percentage of dual-copy BUSCO genes (~ 30%). Although this *A. amphitrite* assembly is a major step forward for the acorn group of barnacles, it is unclear how much of the genome that is captured.

A currently unpublished but publicly available assembly (NCBI GCA_011947565.2) of the stalked barnacle *Pollicipes pollicipes* has a size of 762 Mbp and an N50 value of 109 kbp. The sister species *P. polymerus* (= *Mitella polymerus*) has an experimental genome size of 0.9 Gbp (Bachmann and Rheinsmith 1973), indicating that the *P. pollicipes* assembly may contain a good representation of the genome if the amount of allelic contigs is low (also here DNA was sampled from more than one individual, probably in order to obtain enough DNA for sequencing). Although the rather short contig N50 could constitute a problem in the annotation process, the remarkable scaffold N50 value of 47 Mbp is promising.

A high-quality reference barnacle genome is urgently needed to advance science in this biotechnologically important group of organisms. However, future improvements to the genomes of barnacles will be challenging given the observed high level of genetic diversity, at least for *B. improvisus*, and might require technological innovations/improvements for sequencing, assembly, and annotation. The annotation process for organism groups for which there are no good references to compare with is inherently very difficult. In these cases, genes in many protein families require manual curation to arrive at well-supported gene models. This is true especially for multi-exon genes producing long protein sequences and genes unique to that group of species. Partial and fused genes as well as missing exons in gene-models are common in these types of projects. A way forward could be to use several barnacle species to arrive at a core barnacle proteome.

With the emergence of diploid genome assemblies, we foresee new possibilities in read mapping and accurate allele frequency estimations from population re-sequencing data. While diploid genome assemblies more properly reflect the genome than traditional haploid genome assemblies, new or adapted bioinformatics tools to process re-sequencing data may be needed. In most reference genome assemblies of diploid organisms, the two homologous copies of every chromosome are collapsed and considered as a “mosaic haplotype,” i.e., a “monoploid” (Zhang et al. 2020). While the monoploid reference is simpler to analyze and compare against, it ignores allelic variants that might carry important functions, as has been reported in clinical human studies (Wong et al. 2018; Barroso and McCarthy 2019) and in studies of commercially interesting features of important crops (Shao et al. 2019). Resolving allelic variants will most likely also be essential in understanding and utilizing important properties of marine organisms with biotechnological interest.

The genetic/nucleotide diversity within a species is a key concept in biology and is an important determinant of the species’ evolutionary potential to adapt to new environmental conditions (Ellegren and Galtier 2016). It has been proposed that organisms living in heterogeneous environments will harbor more genetic variation (Flight and Rand 2012; Szulkin et al. 2016; Bisschop et al. 2019), and it was recently suggested that selection on standing genetic variation operates in *S. balanoides* at broad spatial scales to maintain genetic variation by balancing selection in mannose-6-phosphate isomerase (Mpi) (Nunez et al. 2020a). We here report on a strikingly high nucleotide diversity of the *B. improvisus* genome with 5.5% average nucleotide variation in coding regions. Considering that coding regions are generally more conservative than the rest of the genome, the non-coding regions are expected to have higher genetic divergence as we show for the 5′ UTR/promoter region of OctA. However, a complete analysis of general differences in nucleotide diversity between coding and non-coding parts will have to await a better and less fragmented diploid genome assembly. Because coding exons only represent a few percent of animal genomes and intronic and intergenic regions display a much higher variation that also could include insertions and deletions of different sizes, distinguishing between allelic and paralogous regions in *B. improvisus* will likely be hard and create problems when phasing a genome assembly.

It was recently established that the average pairwise nucleotide divergence (π) for the barnacle *S. balanoides* is below 1%, with MPI being one of the most polymorphic genes (π = 2.8%) in this species (Nunez et al. 2020a). Thus, even this extreme case of nucleotide divergence of a coding region in *S. balanoides* is well below the corresponding average divergence we observe in *B. improvisus* (π = 5.5%). It should be noted that *B. improvisus* has been reported to have a high level of genetic diversity in both mitochondrial and nuclear markers in most global populations (Wrange et al. 2016). Nucleotide diversity for cytochrome c oxidase subunit I (COI) in populations of another common fouling barnacle, *A. amphitrite*, was much lower than *B. improvisus* and ranged between 0.7 and 0.9% (Chen et al. 2014). The high nucleotide diversity in *B. improvisus* also stands in sharp contrast to the recently reported very low genetic diversity in the parasitic lantern barnacle *Anelasma squalicola* in a global analysis of diverse populations (Rees et al. 2019).

Furthermore, for the genes/proteins with no amino acid mismatch between the alleles in *B. improvisus*, the pairwise nucleotide diversity was still high (π = 4.2%), indicating that the nucleotide diversity is high also for genes which are under strong purifying selection against non-synonymous substitution. Even more striking is the nucleotide diversity in fourfold degenerate sites in codons, i.e., neutral nucleotide diversity (π4D), where *B. improvisus* displays a diversity of ≈ 20%. This is among the highest nucleotide diversity in coding regions described for any species so far, but in line with the high levels of genetic diversity observed in other marine species, e.g., oyster (Zhang et al. 2012; Leffler et al. 2012). Another extreme case of genetic diversity is found in the split-gill fungus *Schizophyllum commune* for which the neutral nucleotide diversity is 13% in some populations (Baranova et al. 2015). The highest value of neutral nucleotide diversities observed in animals is 16% in the nematode *Caenorhabditis brenneri* (Dey et al. 2013) and 8% in the sea squirt *Ciona savignyi* (Small et al. 2007). *B. improvisus* with its estimate of overall neutral nucleotide diversity of 20% is thus clearly among the hyperdiverse species, i.e., characterized by a neutral nucleotide diversity that exceeds 5% (Cutter et al. 2013).

While some assembly errors cannot be completely ruled out, there is no reason to expect high error rates in our assembly given the sequencing technology used and the obtained assembly read depth. It is of course also possible that a few extreme outliers in π represent divergent gene copies (paralogues or even pseudogenes) rather than alleles, but we consider these cases to be rare. Another note of caution is that our analysis of average nucleotide diversity is based on 66 supposedly single-copy CEGMA genes. This is of course a biased set including proteins that are highly conserved between organisms, and thus possibly more conserved also within species, which could lead to an underestimation of the overall nucleotide diversity for coding regions. On the other hand, the CEGMA genes with split alleles that we identified in *B. improvisus* might be special in that they represent genes that did split-up alleles in the assembly, indicating that they could be more highly divergent genes compared to the rest of the genome. However, it is hard to envisage that our overall estimate of nucleotide diversity would be totally wrong. In particular so for the OctA gene that we analyzed to greater depth, where we found the neutral nucleotide diversity to be in the range 6–12% (Table 3). A complete analysis of the average nucleotide diversity of all coding regions will have to await a high-quality diploid genome assembly where most genes are present, and thus, the average numbers given here for the overall nucleotide diversity should be seen as rough estimates.

The high genetic divergence in *B. improvisus* also has biotechnological implications. Barnacles are the most problematic biofouling marine organisms, where increased fuel consumption and carbon dioxide emissions are examples of problems linked to biofouling of ship hulls. The α-adrenoceptor agonist medetomidine is capable of efficiently inhibiting the settling of barnacles even at nanomolar concentrations (Dahlstrom and Elwing 2006). Octopamine receptors are the invertebrate counterpart of the vertebrate adrenoceptors, and we have earlier shown that the octopamine receptor in *B. improvisus* is encoded by five paralogous genes—one of α-type and four of β-type (Lind et al. 2010). From cloning and functional characterization, it was clear that the most responsive to the antifouling substance medetomidine of the five octopamin receptors is the α-receptor OctA. We have shown that the OctA gene has a high degree of nucleotide divergence even in one single population of *B. improvisus*. The nucleotide divergence could have functional consequences for the octopamine receptor and lead to less efficacy for the antifouling agent medetomidine, thus potentially resulting in that some individuals in the population have higher medetomidine resistance.

Resistance develops quickly in marine organisms, for example in the parasitic copepod *Lepeophtheirus salmonis* (sea lice) that is one of the major concerns in salmon aquaculture (Messmer et al. 2018). Recent estimates of the cost to industry based on this parasite indicate up to 10% of the global value of salmon production. Reductions in the incidence of louse transfer have been largely attributed to louse removal schemes, historically mainly based on treatments with a biocide, the avermectin emamectin benzoate (Messmer et al. 2018). This avermectin binds to glutamate-gated chloride channels, which affects the chloride permeability of neurons (Arena et al. 1995) and leads to death of the parasite. Similar to evolution of antibiotic resistance in human pathogens, reliance on a single antiparasitic drug has increased drug-tolerance toward emamectin in lice populations (Lees et al. 2008). The high-standing genetic variation in OctA gene in *B. improvisus* could similarly provide the ground for development of medetomidine-tolerant populations. It would therefore be advisable to use multiple strategies with different modes of action, e.g., ivermectin (Pinori et al. 2011) or cardiochromene A (Levert et al. 2020) in combination with medetomidine, for long-term resilient antifouling control.

## Conclusion

We believe that the here reported scenario with extremely high genetic diversity and abundant repetitive sequences in *B. improvisus*, and the connected challenges in producing a good reference genome, will be applicable to a wide variety of biotechnologically important marine species with large, out-bred populations having high genetic diversity, e.g., marine invertebrates and fish (Leffler et al. 2012; Plough 2016). Similar challenges were experienced for the establishing of a reference oyster genome, where high-coverage short-read sequences were not sufficient, and they had to revert to an extensive fosmid-based strategy (Zhang et al. 2012) and more recently involving long-read Nanopore sequencing (Wang et al. 2019). Based on our findings, we advise a future *B. improvisus* reference genome project to opt for high-coverage, long-read sequencing of one single individual. Optimally, an inbred individual with a low level of heterozygosity should be used. The high level of standing genetic diversity in a single population of the fouling barnacle *B. improvisus* also indicates that it is advisable to use multiple strategies for antifouling control in order to minimize development of resistance to certain antifouling biocides.

## Supplementary Information

Below is the link to the electronic supplementary material.

Supplementary file1 (PPTX 38 KB) Supplementary information Fig. S1. The frequency of the different size-classes of repeats in *B. improvises*. Repeat Explorer was used to identify high-copy repeats from short sequence reads, which resulted in 1,700 repetitive sequences. The histogram indicates the size-distribution of these repeats (median length 303 bp; min = 90 bp, max = 2361 bp).

Supplementary file2 (XLSX 18 KB) Supplementary information Table S1. Nucleotide diviersity estimates for the 66 CEGMA allelic pairs.

## Data Availability

The unscaffolded assembly is accessible at the SciLifeLab repository (10.17044/scilifelab.14339153).
