# Supplementary figures and images for: Genomic Characterization of the Barnacle Balanus improvisus Reveals Extreme Nucleotide Diversity in Coding Regions
Source: Mar Biotechnol (NY). 2021 May 1;23(3):402–16. doi: 10.1007/s10126-021-10033-8 (PMC8270832; doi:10.1007/s10126-021-10033-8)

## Slide 1
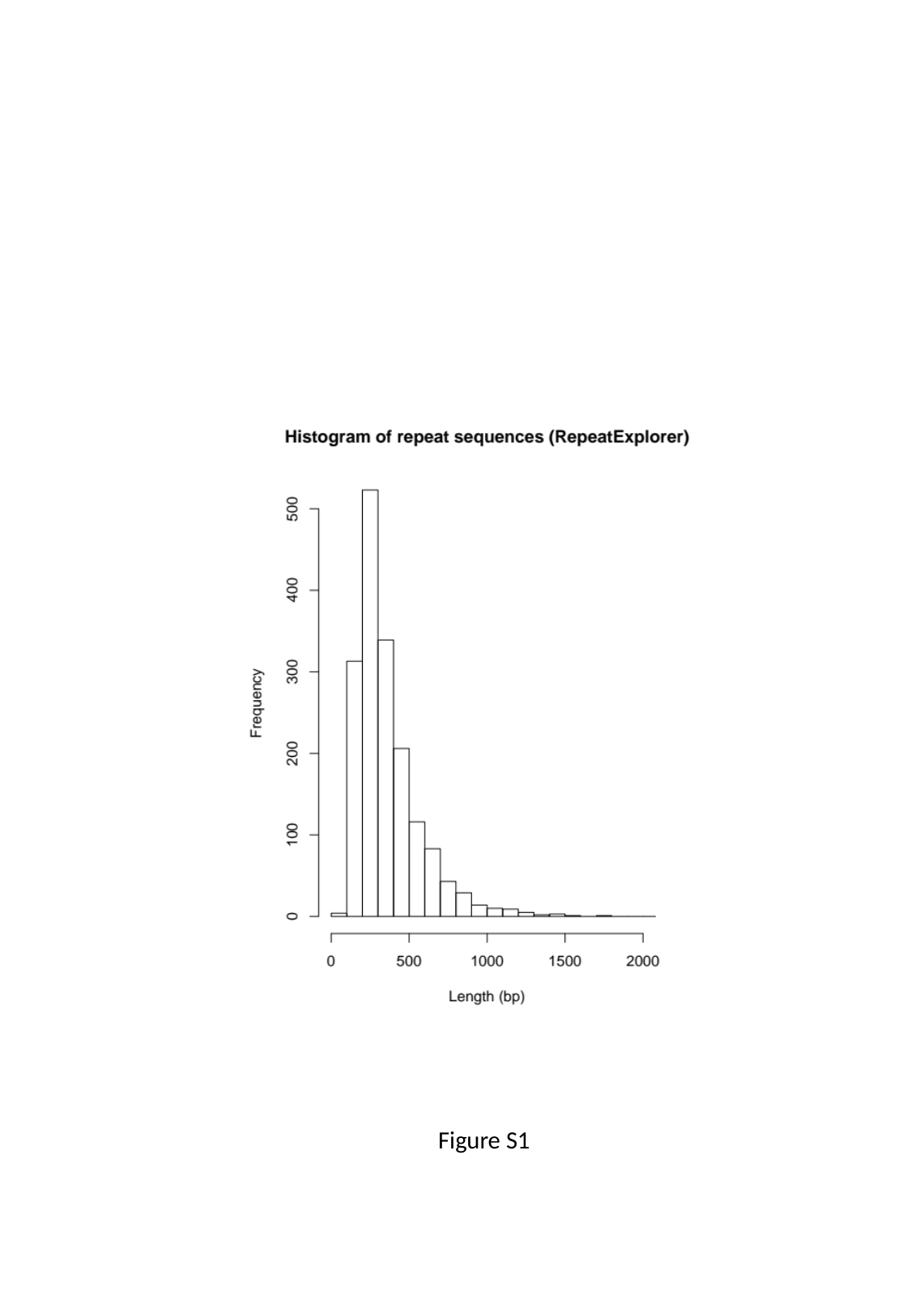

Figure S1

Supplement: Supplementary file 1 — Supplementary file1 (PPTX 38 KB) Supplementary information Fig. S1. The frequency of the different size-classes of repeats in B. improvises. Repeat Explorer was used to identify high-copy repeats from short sequence reads, which resulted in 1,700 repetitive sequences. The histogram indicates the size-distribution of these repeats (median length 303 bp; min = 90 bp, max = 2361 bp). [file 10126_2021_10033_MOESM1_ESM.pptx]
